# Increasing response to a postal survey of sedentary patients – a randomised controlled trial [ISRCTN45665423]

**DOI:** 10.1186/1472-6963-4-31

**Published:** 2004-11-10

**Authors:** Roger A Harrison, Don Cock

**Affiliations:** 1 Research Scientist in Public Health, Directorate of Public Health, Bolton Primary Care Trust, Bolton, UK; 2 Research Associate in Leisure and Tourism, University of Central Lancashire, Preston, UK; 3 Evidence for Population Health Unit, University of Manchester, Manchester, UK

## Abstract

**Background:**

A systematic review identified a range of methods, which can influence response rates. However, analysis specific to a healthcare setting, and in particular, involving people expected to be poor responders, was missing, We examined the effect of pre-warning letters on response rates to a postal survey of sedentary patients whom we expected a low rate of response.

**Methods:**

Participants were randomised to receive a pre-warning letter or no pre-warning letter, seven days before sending the main questionnaire. The main questionnaire included a covering letter and pre-paid return envelope. After seven days, non-responders were sent a reminder letter and seven days later, another reminder letter with a further copy of the questionnaire and return envelope.

**Results:**

627 adults, with a mean age of 48 years (SD 13, range 18 to 78) of whom 69.2% (434/627) were women, were randomised. 49.0% (307/627) of patients were allocated to receive a pre-warning letter and 51.0% (320/627) no pre-warning letter, seven days in advance of posting the main questionnaire. The final response rate to the main questionnaire was 30.0% (92/307) amongst those sent a pre-warning letter and 20.9% (67/320) not sent a pre-warning letter, with an adjusted odds ratio of 1.60 (95% CI 1.1, 2.30).

**Conclusions:**

The relatively low cost method of sending a pre-warning letter had a modest impact on increasing response rates to a postal questionnaire sent to a group of patients for whom a low response rate was anticipated. Investigators should consider incorporating this simple intervention when conducting postal surveys, to reduce the potential for nonresponse bias and to increase the study power. Methods other than postal surveys may be needed however when a low response rate to postal surveys is likely.

## Background

Postal surveys are routinely used to obtain information from patients and groups within the general population, over a range of topics. Postal surveys are a cost-efficient method compared with intensive methods such as face-to-face interviews and capable of obtaining systematically, information on many thousands of people. A key quality component of postal surveys relates to the number of people sampled, and the proportion returning a completed useable questionnaire [1]. Lower response rates can reduce the statistical power of the study, and mask statistically significant relationships, which 'truly' exist within the population studied. Responders may also be different to non-responders. This can introduce bias into the survey findings, if the decision to respond (or not) relates to the outcome being analysed within the survey, thereby reducing generalisability to the initial reference population [2].

Many studies conclude that non-responders in surveys and other epidemiological studies can differ to responders with respect to a range of specific health, lifestyle and social variables. Non-responders have been found to differ with respect to their sex, age, race, social class, home circumstances, education, and healthy lifestyle behaviours [3-7]. They can also differ in terms of existing health and healthcare utilisation [8-11] with differences extending through to higher rates of mortality [12] compared with responders. However, it can be difficult to make clear conclusions about the characteristics of non-responders in surveys and other types of studies, as factors such as the purpose of the study and the way in which it was carried out, will no-doubt have some effect. Furthermore, differences have not always been found between responders and non-responders, at least in terms of what factors were assessed, and nonresponse will not always affect estimates of prevalence [13-15]. Nevertheless, nonresponse bias should always be considered a possibility [16,17]. Moreover, there is evidence that in general, response rates to postal questionnaires are falling, [18], making this topic worthy of continued investigation. There is however, no agreed level of acceptable response in postal surveys [19-21].

A systematic review [22] found a number of factors associated with postal questionnaires can influence the likelihood of response. This included providing incentives; questionnaire length and appearance, method of delivery, method for return, if any pre-warning/contact was given, the content and layout of the questions, through to the origin/sponsor of the questionnaire and how it was communicated. The review found that monetary incentives, recorded delivery and using an 'interesting' questionnaire were the three strongest influences on response rates. Statistical heterogeneity was found for all of these factors, thus limiting the extent that pooling of results was viable. Moreover, only a third of studies were from medical/epidemiological/healthcare journals and with no distinction between studies of patients or of staff and subgroup analyses were absent. As such, it is difficult to generalise the findings from the review to particular groups of patients in a healthcare setting.

In the current study, we carried out a randomised controlled trial to examine the effect of a pre-warning letter on response rates to a postal questionnaire. The questionnaire was sent to patients who had previously been referred to a community based exercise referral scheme because of a sedentary lifestyle and sought information on the quality of the service offered. An earlier study [23] of a similar population suggested poor response rates could be a problem. With a limited budget, we were unable to offer financial incentives, as suggested in the review by Edwards et al [22], but wanted to explore the suggestion that pre-warning letters might increase final response.

## Methods

The sample consisted of patients who had been referred to a community based exercise referral scheme during the past 12 months, identified from the service register. Patients were referred by a primary care practitioner because of concerns about their sedentary behaviour and its impact on their health. The questionnaire formed part of a project examining the relationship between patient service-expectations and service outcomes.

We examined the effect of a pre-warning letter, posted to patients seven-days before sending the main questionnaire, compared with no pre-warning letter. The pre-warning letter was printed on one-side of letter-headed paper, and informed the respondent that a survey would be sent to them within the next few days. It informed them about the purpose of the survey and the importance of it being completed and returned.

The main questionnaire was sent with a covering letter and a pre-paid business franked addressed envelope for its return [24]. A standard reminder letter was sent to all non-responders seven days after posting the questionnaire, and after a further seven days, persistent non-responders were sent a further copy of the questionnaire, with a standard letter and return envelope. Randomisation was done using computer generated random numbers, and stratifying by age and sex. Participants remained unaware as to group allocation.

The primary outcome was the final response rate to postal questionnaires after sending all reminder letters. This was calculated at least 6 weeks after sending the initial questionnaire, to allow for late responders. Differences in proportions between groups were examined using Pearson's chi-square test and logistic regression to adjust for age and sex with 95% confidence intervals (95% CI). To observe a difference of at least 10% between trial arms required 752 participants, based on a return rate of 60% in the control groups, with 80% power. Approval for the study was received in advance from the local research ethics committee and the research governance committee.

## Results

**Figure 1:**
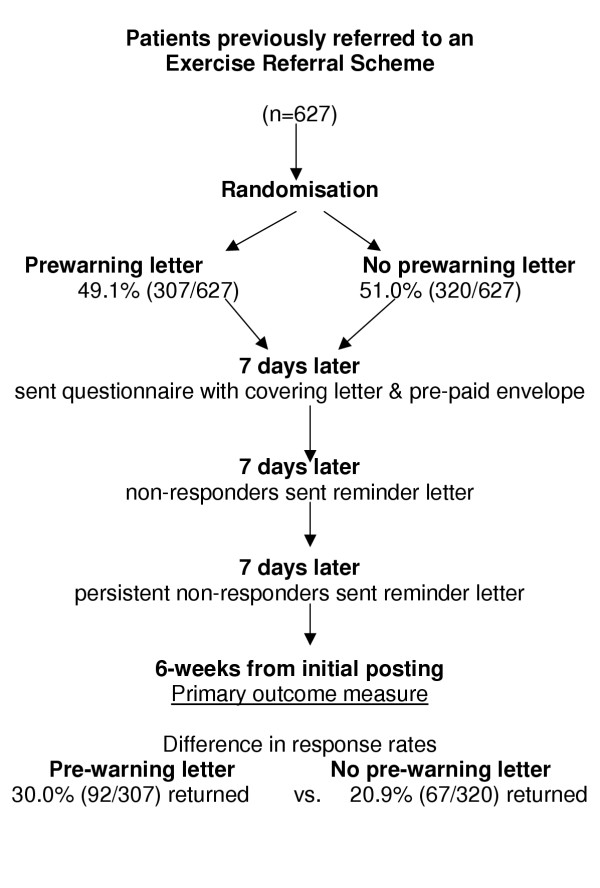
Flow diagram of the randomised controlled trial.

The number of patients referred to the exercise referral scheme in the past year with complete name and address information was 627. Their mean age was 48 years (SD 13, range 18 to 78) and 69.2% (434/627) were women. Randomisation allocated 49.0% (307/627) of patients to receive a pre-warning letter and 51.0% (320/627) no pre-warning letter (Figure 1). The two groups were balanced with respect to sex (66.8% female in the pre-warning group compared with 71.6% in the control group) and age (mean 48.7 years, SD 13.3, in the pre-warning group compared with mean 47.6 years, SD 13.9 in the control group).

**Figure 2:**
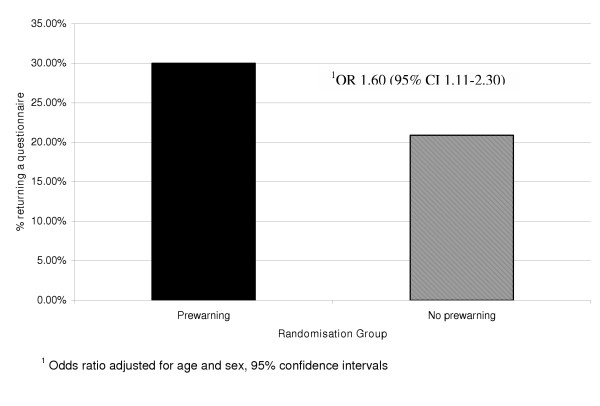
Effect of pre-warning on final questionnaire response rates.

The final response rate to the postal survey, after completing two stages of follow-up was 25.4% (159/627). In the pre-warning group, the response rate was 30.0% (92/307) compared with 20.9% (67/320) in the control group (χ^2 ^6.75, p = 0.009) (Figure 2). Thus giving a difference between the two groups of 9.1% (95% CI for risk difference, 2.2% to 15.8%) (Figure 2). In a logistic regression model, the pre-warning letter increased the odds of returning the questionnaire by 1.61 (95% confidence interval 1.12, 2.32) and this was not altered after adjusting for age (in years) or sex (OR^adj age sex ^1.60, 95% CI 1.11, 2.30).

## Conclusions

Sending a pre-warning letter seven days in advance of mailing out a postal questionnaire had a modest impact on increasing final response rates by almost 10%, with a relative 43% increase compared with sending no pre-warning letter. Patients in our study were selected on the basis of a previous referral to a community based exercise referral scheme, and the questionnaire sent to them sought information about their perceptions of the service quality. An earlier trial examining the impact of this service on a similar group of patients, in terms of increasing physical activity, had achieved average response rates of 60% [23]. The much lower than expected response rate in the current study could have been influenced by the topic and purpose of the questionnaire along with the layout of the questionnaire [22].

Our findings are consistent with the evidence from a systematic review [22] that pre-contact can lead to an increased odds of response by as much as 50%. Our study confirms that this benefit extends to groups of sedentary patients known in advance, to be reluctant to reply. Pre-warning letters are simple to administer and relatively low cost compared with more labour intensive methods to increase response rates, such as telephone reminders or face-to-face visits. Moreover, this intervention method does not require any additional information or administrative systems over and above those required for sending the main questionnaire. Give that the response rate in the intervention group still remained low, at 30%, we may need to consider alternative approaches to postal questionnaires to obtain information from this group of patients, particularly if non-response could be associated with the outcomes examined within the survey – in this case, the patient experience of the exercise referral service.

## Competing interests

The authors declare that they have no competing interests.

## Authors' contributions

RAH conceived the study design, drafted the main protocol, carried out randomisation, analysed the results and wrote the first draft of the paper.

DC was responsible for completing the study, for data entry, for assisting with data analysis and significant comments on the manuscript.

## Pre-publication history

The pre-publication history for this paper can be accessed here:
